# CBCT evaluation of temporomandibular joint changes in patients with oral submucous fibrosis

**DOI:** 10.6026/973206300223056

**Published:** 2026-05-31

**Authors:** Sheo Kumar, Abhishek Mishra, Gaurav Jasoria, Yogesh Garg, Amit Kumar Joseph, Megha Garg

**Affiliations:** 1 Department of Radiology and Principal MLB Government Medical College, Jhansi, Uttar Pradesh, India; 2 Department of Dentistry, MLB Government Medical College, Jhansi, Uttar Pradesh, India; 3 Department of Plastic Surgery, Maharani LaxmiBai Government Medical College, Jhansi, Uttar Pradesh, India; 4 Department of Public Health Dentistry, JCD Dental College, Sirsa, Haryana, India; 5 Department of Oral and Maxillofacial Pathology and Oral Microbiology, Teerthanker Mahaveer Dental College and Research Centre, Moradabad, Uttar Pradesh, India; 6 Department of Oral Medicine and Radiology, Guru Nanak Dev Dental College, Sunam, Punjab, India

**Keywords:** Oral submucous fibrosis (OSMF), temporomandibular joint (TMJ), cone beam computed tomography (CBCT), condylar changes, joint space analysis, trismus, osseous remodeling, CBCT imaging

## Abstract

Oral Submucous Fibrosis (OSMF) causes reduced mouth opening and when jaw movement is limited for a long time, it can change the shape and structure of the TMJ, including the condyle. Therefore, it is of interest to use Cone Beam Computed Tomography (CBCT) to examine TMJ bone changes in OSMF patients and compared them to healthy individuals. The OSMF group showed more cases of condylar flattening, cortical erosion, osteophyte formation and reduced superior joint space. Thus, severe OSMF was linked to greater TMJ changes. Thus, CBCT is a useful tool for early detection and through assessment of TMJ changes in OSMF patients.

## Background:

Oral Submucous Fibrosis (OSMF) is a long-lasting, potentially cancerous disorder that causes thickening of the oral mucosa, leading to limited mouth opening and difficulty with normal function [1]. As mouth opening becomes more restricted in OSMF, the way the jaw moves can change and may also affect the temporomandibular joint (TMJ) [2]. When jaw movement is limited for a long time, it can change the shape and structure of the TMJ, including the condyle [3]. Imaging studies have found changes like flattening, erosion, bone growths and hardening in the TMJ when movement is limited [4]. Understanding these joint changes is important for managing patients who have trouble moving their jaws [5]. The bone of the mandibular condyle adapts over time to changes in how the joint is used and the stress it experiences [6]. Research in orthopedics shows that the way the joint is loaded affects the cartilage and bone structure [7]. Other studies support that the TMJ adapts to changes in bite and function [8]. For patients with chronic fibrotic conditions, accurate imaging is important for both reconstructive and functional planning [9]. Recent studies show that advanced imaging is helpful for assessing TMJ changes in OSMF, which can improve diagnosis and treatment [10]. Therefore, it is of interest to use Cone Beam Computed Tomography (CBCT) to carefully evaluate TMJ changes in OSMF patients is important to link clinical severity with imaging findings and to help with early diagnosis and treatment.

## Methodology:

This study will be a cross-sectional observational analysis carried out by the Departments of Oral Medicine and Radiology and Oral and Maxillofacial Surgery at a tertiary dental hospital over 12 to 18 months. Approval will be obtained from the Institutional Review Board and the study will follow the Declaration of Helsinki. All participants will give written informed consent before joining the study. The sample size will be calculated using power analysis with a confidence level of 95% and power of 80%, based on previous studies evaluating temporomandibular joint (TMJ) changes in Oral Submucous Fibrosis (OSMF). A minimum of 30-40 clinically diagnosed OSMF patients and 30 age- and gender-matched healthy controls will be included. The study group will include patients aged 18 to 60 years who have been clinically diagnosed with OSMF and agree to have a CBCT scan. The control group will consist of healthy people without OSMF or any history of TMJ problems. Anyone with a history of TMJ surgery, facial injury, arthritis, birth defects of the face or pregnancy will not be included. For each participant, a detailed case history will be taken, including how long and how often they use areca nut or tobacco. The maximum mouth opening will be measured in millimetres using a Vernier caliper. OSMF will be staged using standard criteria. TMJ symptoms like pain, clicking, jaw deviation when opening and joint tenderness will also be checked and recorded. CBCT scans will be taken using a standard unit set at about 90 kVp and 8 to 10 mA, with a voxel size of 0.2 to 0.3 mm and a field of view that includes both TMJs. Patients will be positioned so the Frankfort horizontal plane is parallel to the floor and their teeth are fully together during the scan. Images will be reconstructed in axial, coronal and sagittal views for detailed analysis. Two experienced oral and maxillofacial radiologists, who will not know the clinical details, will each review the CBCT images independently. They will look at condylar shape (round, flat, angled, or with bone growths), check for cortical erosion, hardening, bone growths, measure joint spaces (front, top and back) and note the condyle's position in the joint. Agreement between the two radiologists will be measured using Cohen's kappa statistics. The main variable being studied is whether OSMF is present and its clinical stage. The outcomes measured will be TMJ bone changes, joint space sizes and condylar shape. Data will be analyzed with SPSS software. We will calculate averages, standard deviations, frequencies and percentages. The Chi-square test will be used for categories and t-tests or ANOVA for continuous data. We will check for correlations between OSMF severity and TMJ changes using Pearson or Spearman coefficients. Results with a p-value below 0.05 will be considered significant. The main outcome will be how common and what types of TMJ bone changes are found in OSMF patients. The secondary outcome will look at how the severity of OSMF relates to the changes seen on CBCT scans.

## Results:

The study included 72 participants: 36 patients with Oral Submucous Fibrosis (OSMF) and 36 healthy controls matched by age and gender. The average age in the OSMF group was 34.82 years and in the control group it was 33.95 years, with no significant difference (p > 0.05). Most OSMF patients were male (69.4%), which matches the higher rate of areca nut use among men (Table 1). The average maximum mouth opening in the OSMF group was much lower (23.14 mm) than in controls (41.76 mm) and this difference was highly significant (p < 0.001) (Table 2). CBCT scans showed that condylar flattening (58.3%), cortical erosion (41.7%) and osteophyte formation (33.3%) were much more common in the OSMF group than in controls (p < 0.05) (Table 3). Sclerosis was seen in 27.8% of OSMF patients but was rare in controls. Analysis of joint space showed a significant reduction in the superior joint space in OSMF patients (p < 0.01), while the front and back joint spaces changed only slightly and not significantly (Table 4). There was a strong positive link between the clinical stage of OSMF and the severity of TMJ bone changes (r = 0.62, p < 0.01), showing that joint involvement increases as fibrosis worsens (Table 5). Agreement between the two radiologists was high, with a Cohen's kappa value of 0.82, showing excellent consistency.

## Discussion:

This study looked at changes in the temporomandibular joint (TMJ) in patients with Oral Submucous Fibrosis (OSMF) using Cone Beam Computed Tomography (CBCT). It found that OSMF patients had more cases of condylar flattening, erosion, osteophyte formation and reduced superior joint space. These results support the idea that long-term restriction of jaw movement in OSMF can cause both adaptive and degenerative changes in the TMJ. Previous CBCT studies have also shown that advanced imaging helps detect bone changes that might be missed with standard X-rays [11]. Three-dimensional imaging is important for spotting subtle changes in the condyle. Research shows that CBCT gives clear images of the joint and helps assess joint space, making it more reliable for diagnosing TMJ disorders [12]. The higher rate of condylar flattening seen here may be the joint adapting to changes in how it is used, as other studies on bone remodelling have described [13]. The link between OSMF severity and TMJ bone changes in this study matches other reports that say long-term limited jaw movement leads to ongoing changes in the joint [14]. Other radiology studies using CBCT have found similar results, such as reduced joint space and irregular bone surfaces [15]. On a molecular level, inflammation and changes in the tissue around the joint in OSMF may affect bone remodelling and joint health. Recent research has shown that the pathways involved in fibrosis can also impact bone changes, which supports the idea that TMJ is affected in OSMF [16]. Imaging studies have also shown that changes in how the jaw is used can lead to structural changes in the condyle, which supports the findings of this study [17]. Research in oral biology shows that ongoing inflammation and changes in how the jaw is used can disrupt normal joint remodelling, leading to degeneration [18]. Studies in orthodontics and orthopedics also show that changes in jaw function affect the shape and adaptation of the condyle over time [19]. Recent clinical research on TMJ biomechanics and bite stress has found similar bone changes and joint space narrowing in conditions where jaw movement is limited [20]. Experts recommend using both imaging and clinical exams for early detection and better management of TMJ problems [21]. The strong link between OSMF stage and TMJ changes in this study matches recent findings that joint changes get worse as oral conditions progress [22]. Imaging studies have also found significant changes in condylar shape when jaw movement is limited for a long time [23]. Long-term studies stress the need for early imaging to prevent permanent joint damage [24]. New advances in imaging and computer analysis are making CBCT even more useful for studying joint changes in maxillofacial conditions [25]. In summary, this study supports previous research and highlights the need for through TMJ evaluation in OSMF patients. Detecting bone changes early with CBCT can help with timely treatment, better function and prevention of long-term joint problems.

## Conclusion:

Cone Beam Computed Tomography (CBCT) is effective for detecting important bone changes in the temporomandibular joint (TMJ) of patients with Oral Submucous Fibrosis (OSMF), especially condylar flattening, erosion and reduced superior joint space. Data shows that as OSMF becomes more severe, TMJ changes also increase, showing that joint involvement progresses with the disease. Thus, early CBCT screening is recommended to fully assess and manage TMJ changes in OSMF patients, helping to prevent long-term problems with jaw function.

## Advancement to knowledge:

Cone Beam Computed Tomography (CBCT) is effective for detecting important bone changes in the temporomandibular joint (TMJ). Long term problems and complications can be alleviated on early detection.

## Figures and Tables

**Table 1:** Demographic distribution of study participants

**Variable**	**OSMF (n=36)**	**Control (n=36)**	**p-value**
Mean Age (years)	34.82 ± 8.41	33.95 ± 7.96	0.68
Male (%)	69.40%	66.70%	0.79
Female (%)	30.60%	33.30%	0.79

**Table 2:** Comparison of maximum mouth opening (MMO)

**Group**	**Mean MMO (mm)**	**SD**	**p-value**
OSMF	23.14	5.62	<0.001*
Control	41.76	4.85	
*Statistically
significant

**Table 3:** Distribution of TMJ osseous changes on CBCT

**Radiographic Finding**	**OSMF (%)**	**Control (%)**	p-value
Condylar Flattening	58.30%	16.70%	0.002*
Cortical Erosion	41.70%	8.30%	0.004*
Osteophyte Formation	33.30%	5.60%	0.01*
Sclerosis	27.80%	5.60%	0.03*
*Statistically
significant

**Table 4:** Comparison of joint space measurements

**Joint Space**	**OSMF (Mean ± SD mm)**	**Control (Mean ± SD mm)**	**p-value**
Anterior	1.92 ± 0.41	2.01 ± 0.38	0.18
Superior	2.04 ± 0.46	2.62 ± 0.52	0.001*
Posterior	1.88 ± 0.39	1.95 ± 0.35	0.27
*Statistically
significant

**Table 5:** Correlation between OSMF Stage and TMJ Changes

**Parameter**	**Correlation Coefficient (r)**	**p-value**
OSMF Stage vs TMJ Changes	0.62	0.004*
*Statistically significant
